# Children under 10 years of age were more affected by the 2018/19 influenza A(H1N1)pdm09 epidemic in Canada: ‎possible cohort effect following the 2009 influenza pandemic

**DOI:** 10.2807/1560-7917.ES.2019.24.15.1900104

**Published:** 2019-04-11

**Authors:** Danuta M Skowronski, Siobhan Leir, Gaston De Serres, Michelle Murti, James A Dickinson, Anne-Luise Winter, Romy Olsha, Matthew A Croxen, Steven J Drews, Hugues Charest, Christine Martineau, Suzana Sabaiduc, Nathalie Bastien, Yan Li, Martin Petric, Agatha Jassem, Mel Krajden, Jonathan B Gubbay

**Affiliations:** 1 British Columbia Centre for Disease Control, Vancouver, Canada; 2 University of British Columbia, Vancouver, Canada; 3 Institut National de Santé Publique du Québec, Quebec, Canada; 4 Laval University, Quebec, Canada; 5 Centre Hospitalier Universitaire de Québec, Quebec, Canada; 6 Public Health Ontario, Toronto, Canada; 7 University of Toronto, Toronto, Canada; 8 University of Calgary, Calgary, Canada; 9 Provincial Laboratory for Public Health, Edmonton, Canada; 10 University of Alberta, Edmonton, Canada; 11 National Microbiology Laboratory, Public Health Agency of Canada, Winnipeg, Canada

**Keywords:** influenza, A(H1N1)pdm09, A(H3N2), age, epidemic, pandemic, Canada, air-borne infections, viral infections, influenza, influenza-like illness, ILI, influenza virus, laboratory surveillance, sentinel surveillance, epidemiology, laboratory

## Abstract

**Introduction:**

Findings from the community-based Canadian Sentinel Practitioner Surveillance Network (SPSN) suggest children were more affected by the 2018/19 influenza A(H1N1)pdm09 epidemic.

**Aim:**

To compare the age distribution of A(H1N1)pdm09 cases in 2018/19 to prior seasonal influenza epidemics in Canada.

**Methods:**

The age distribution of unvaccinated influenza A(H1N1)pdm09 cases and test-negative controls were compared across A(H1N1)pdm09-dominant epidemics in 2018/19, 2015/16 and 2013/14 and with the general population of SPSN provinces. Similar comparisons were undertaken for influenza A(H3N2)-dominant epidemics.

**Results:**

In 2018/19, more influenza A(H1N1)pdm09 cases were under 10 years old than controls (29% vs 16%; p < 0.001). In particular, children aged 5–9 years comprised 14% of cases, greater than their contribution to controls (4%) or the general population (5%) and at least twice their contribution in 2015/16 (7%; p < 0.001) or 2013/14 (5%; p < 0.001). Conversely, children aged 10–19 years (11% of the population) were under-represented among A(H1N1)pdm09 cases versus controls in 2018/19 (7% vs 12%; p < 0.001), 2015/16 (7% vs 13%; p < 0.001) and 2013/14 (9% vs 12%; p = 0.12).

**Conclusion:**

Children under 10 years old contributed more to outpatient A(H1N1)pdm09 medical visits in 2018/19 than prior seasonal epidemics in Canada. In 2018/19, all children under 10 years old were born after the 2009 A(H1N1)pdm09 pandemic and therefore lacked pandemic-induced immunity. In addition, more than half those born after 2009 now attend school (i.e. 5–9-year-olds), a socio-behavioural context that may enhance transmission and did not apply during prior A(H1N1)pdm09 epidemics.

## Introduction

The 2018/19 seasonal influenza epidemic in the northern hemisphere has primarily been due to influenza A viruses with fewer influenza B detections than usual [1-3]. In Canada, among influenza A viruses subtyped, the majority (> 80% as at week 11) have been influenza A(H1N1)pdm09 [1]. Influenza A(H1N1)pdm09 viruses have also predominated elsewhere, including the United States (US) [2] and Europe [3,4], but with more variable contribution by influenza A(H3N2) subtype viruses. The last influenza A(H1N1)pdm09-dominant epidemics in Canada occurred in 2013/14 [1,5] and 2015/16 [1,6]; A(H3N2) subtype viruses predominated in 2014/15 [1,7], 2016/17 [1,8] and 2017/18 [1,9].

The 2018/19 influenza A(H1N1)pdm09 epidemic in Canada began early in week 43, with paediatric hospitalisations above the seasonal norm as early as week 45 and national surveillance indicators showing the epidemic peak during week 52 2018 [1]. In a rapid communication, published 24 January 2019, the community-based Canadian Sentinel Practitioner Surveillance Network (SPSN) reported early estimates of influenza vaccine effectiveness (VE) for the 2018/19 season [10]. Substantial VE against medically-attended outpatient influenza A(H1N1)pdm09 illness was reported for all age groups and particularly for children. Vaccine protection against influenza A(H1N1)pdm09 was also subsequently reported among outpatients in the US [11], and Europe [12] and from Hong Kong among children requiring inpatient care [13].

The Canadian SPSN interim report, which captured cases as at week 2 2019, suggested that children aged less than 10 years were disproportionately affected by the 2018/19 influenza A(H1N1)pdm09 epidemic [10]. Drawing on historical datasets of the Canadian SPSN since the 2013/14 seasonal influenza A(H1N1)pdm09 epidemic, we examine and compare this age-related surveillance signal in more detail and inclusive of additional weeks spanning the tail end of the 2018/19 influenza A(H1N1)pdm09 epidemic.

## Methods

### Study participants and protocol

As described in prior SPSN publications [5-10], nasal/nasopharyngeal specimens and epidemiological data were collected from patients presenting to community-based sentinel practitioners in the four most populous provinces of Canada: Alberta, British Columbia, Ontario and Quebec. Eligible patients met a standardised case definition for influenza-like illness (ILI) consisting of acute onset of self-reported fever and cough and at least one other symptom including sore throat, myalgia, arthralgia or prostration [5-10]. The same ILI criteria applied each season and to all age groups, except that fever was not a requirement for elderly adults aged 65 years and older. All specimens were tested for influenza type and subtype by real-time RT-PCR assays at provincial public health laboratories; subtyping information was available for ≥ 95% of influenza A viruses each season [5-10].

The current study is restricted to specimens collected from patients presenting within 7 days of ILI onset between 1 November and 30 April each seasonal influenza epidemic, except 2018/19 for which collection dates spanned from 1 November to specimens available as at 18 March 2019 [5-10] (Supplementary Figure S1). Cases tested positive for the dominant influenza A subtype of a given season; controls tested negative for any influenza virus. Patients without available influenza A subtype information were excluded. To assess age distribution in the absence of vaccine effects, analyses were restricted to unvaccinated patients aged 1 year or older. Unvaccinated patients were those self-reporting no influenza vaccine receipt (or as reported by their parent/guardian) in the season during which their specimen was collected.

### Ethical statement

Although conducted as a surveillance initiative, the Canadian SPSN follows a study protocol that includes obtaining verbal consent from patients (or their parent/guardian). During the study period the protocol was approved by the following ethics review committees in participating provinces: University of Calgary, Calgary, Alberta; University of British Columbia, Vancouver, British Columbia; University of Toronto (2013/14–2016/17), University Health Network (2013/14–2016/17), and Public Health Ontario (2017/18–2018/19), Toronto, Ontario; and Comité d’éthique de santé publique, Québec.

### Age-related analyses

The age distribution of unvaccinated influenza A(H1N1)pdm09 cases and unvaccinated controls in 2018/19 was primarily compared with prior influenza A(H1N1)pdm09-dominant seasonal epidemics (i.e. 2013/14 and 2015/16) [5,6]. Similar comparisons were undertaken for influenza A(H3N2)-dominant epidemics (i.e. 2014/15, 2016/17 and 2017/18) [7-9]. Age-related analyses and comparisons by season included:

(i) The percentage of ILI specimens testing positive for the specified influenza A subtype, by age group. Age groups included: children aged 1–4 years; 5–9 years and 10–19 years; and adults aged 20–49 years; 50–64 years; and 65 years and older (elderly adults). Percentage positivity estimates were accompanied by 95% confidence intervals (CIs) and compared by chi-squared analysis.

(ii) The percentage distribution of unvaccinated cases and controls by single year of age. Percentage histograms displayed the percentage of all cases (of the specified influenza A subtype) that belonged to a given age in years such that percentages sum 100% across the age range each season. The percentage distribution of influenza test-negative controls was superimposed upon the same season-specific plots to standardise for potential age-related sampling variation. The median age of cases and controls was compared within and across seasons by the Wilcoxon rank-sum test.

(iii) The percentage distribution of unvaccinated cases and controls by age group. Proportions were accompanied by 95% CIs and compared by chi-squared analysis. Case/control distributions by age group were also compared with the general population of SPSN provinces (2018 data) (Supplementary Table S1) [14].

## Results

### Percentage positivity by age category

In Table 1, among specimens collected from unvaccinated patients with ILI, the age group with the highest influenza A test-positivity was children aged 5–9 years in 2018/19 (73% A(H1N1)pdm09 positive). This positivity rate was not only significantly higher compared to the same age group during prior influenza A(H1N1)pdm09 epidemics in 2015/16 (34%; p <  0.001) and 2013/14 (31%; p < 0.001), but it was also significantly higher compared to any other age group in any other seasonal epidemic assessed (all p values < 0.005).

**Table 1:** Percentage of ILI specimens testing positive for the specified influenza A subtype, by age group, Canadian Sentinel Practitioner Surveillance Network (SPSN), seasonal influenza epidemics 2013/14–2018/19

Subtype and Season	Age group																							
	1–4 years				5–9 years				10–19 years				20–49 years				50–64 years				≥ 65 years			
	n	N	%	95% CI	n	N	%	95% CI	n	N	%	95% CI	n	N	%	95% CI	n	N	%	95% CI	n	N	%	95% CI
**Seasonal influenza A(H1N1)pdm09 epidemics**																								
2013/14^a^	26	85	**31**	21–42	19	61	**31**	20–44	32	113	**28**	20–38	201	561	**36**	32–40	76	181	**42**	35–50	12	48	**25**	14–40
2015/16^b^	50	135	**37**	29–46	38	111	**34**	26–44	38	160	**24**	17–31	280	718	**39**	35–43	97	251	**39**	33–45	22	70	**31**	21–44
2018/19^c^	125	244	**51**	45–58	124	171	**73**	65–79	57	188	**30**	24–37	389	876	**44**	41–48	131	331	**40**	34–45	32	98	**33**	24–43
**Seasonal influenza A(H3N2) epidemics**																								
2014/15^d^	25	107	**23**	16–33	38	90	**42**	32–53	59	166	**36**	28–43	166	519	**32**	28–36	67	209	**32**	26–39	25	59	**42**	30–56
2016/17^e^	29	130	**22**	16–30	49	91	**54**	43–64	97	206	**47**	40–54	211	597	**35**	32–39	91	235	**39**	33–45	44	102	**43**	33–53
2017/18^f^	21	83	**25**	16–36	32	97	**33**	24–43	50	202	**25**	19–31	197	775	**25**	22–29	98	307	**32**	27–38	40	113	**35**	27–45

CI: confidence interval; ILI: influenza-like illness; n: number of specimens collected from unvaccinated patients that tested positive for the specified influenza A subtype that predominated during the specified season; N: total number of included specimens collected from unvaccinated patients with ILI and meeting other eligibility criteria.

a 2013/14 span of specimen collection dates for unvaccinated cases (n = 366): 6 November 2013 to 3 April 2014; for unvaccinated controls (n = 683): 1 November 2013 to 29 April 2014.

^b^ 2015/16 span of specimen collection dates for unvaccinated cases (n = 525): 9 December 2015 to 28 April 2016; for unvaccinated controls (n = 920): 1 November 2015 to 30 April 2016.

^c^ 2018/19 span of specimen collection dates for unvaccinated cases (n = 858): 1 November 2018 to 14 March 2019; for unvaccinated controls (n = 1,050): 1 November 2018 to 18 March 2019.

^d^ 2014/15 span of specimen collection dates for unvaccinated cases (n = 380): 5 November 2014 to 1 April 2015; for unvaccinated controls (n = 770): 3 November 2014 to 30 April 2015.

^e^ 2016/17 span of specimen collection dates for unvaccinated cases (n = 521): 1 November 2016 to 27 April 2017; for unvaccinated controls (n = 840): 1 November 2016 to 29 April 2017.

^f^ 2017/18 span of specimen collection dates for unvaccinated cases (n = 438): 9 November 2017 to 26 April 2018; for unvaccinated controls (n = 1,139): 1 November 2017 to 30 April 2018.

The % columns indicate the percentage of total specimens meeting eligibility criteria (N) that were test-positive (n) for the specified influenza A subtype, by age group and season; these % values are shown in bold for clarity.

Higher influenza A(H1N1)pdm09 test-positivity was also observed among children aged 1-4 years in 2018/19 compared to 2015/16 and 2013/14 (51% vs 37%; p = 0.008 and 31%; p = 0.001, respectively) and among adults 20-49 years old (44% vs 39%; p = 0.03 and 36%; p = 0.001, respectively), but these differences were less striking. Other age groups showed no significant differences across these seasons.

The next highest percentage influenza A positivity (54%) also involved children aged 5–9 years but during the influenza A(H3N2) epidemic in 2016/17 (p = 0.002 compared with 2018/19). Children aged 10–19 years also had higher influenza A(H3N2) positivity rates in 2016/17 (47%), greater than for any other season for that age group (36% in 2014/15 (p = 0.03); p < 0.005 any other seasons’ comparisons). Among those aged 65 years and older, influenza test-positivity was also generally higher during A(H3N2) vs A(H1N1)pdm09 epidemics, marginally significant in comparing positivity across combined A(H3N2) vs A(H1N1)pdm09 epidemics (40% vs 31%; p = 0.03) and between 2016/17 vs 2013/14 (43% vs 25%; p = 0.03) .

### Percentage distribution by age in years

**Figure 1:**
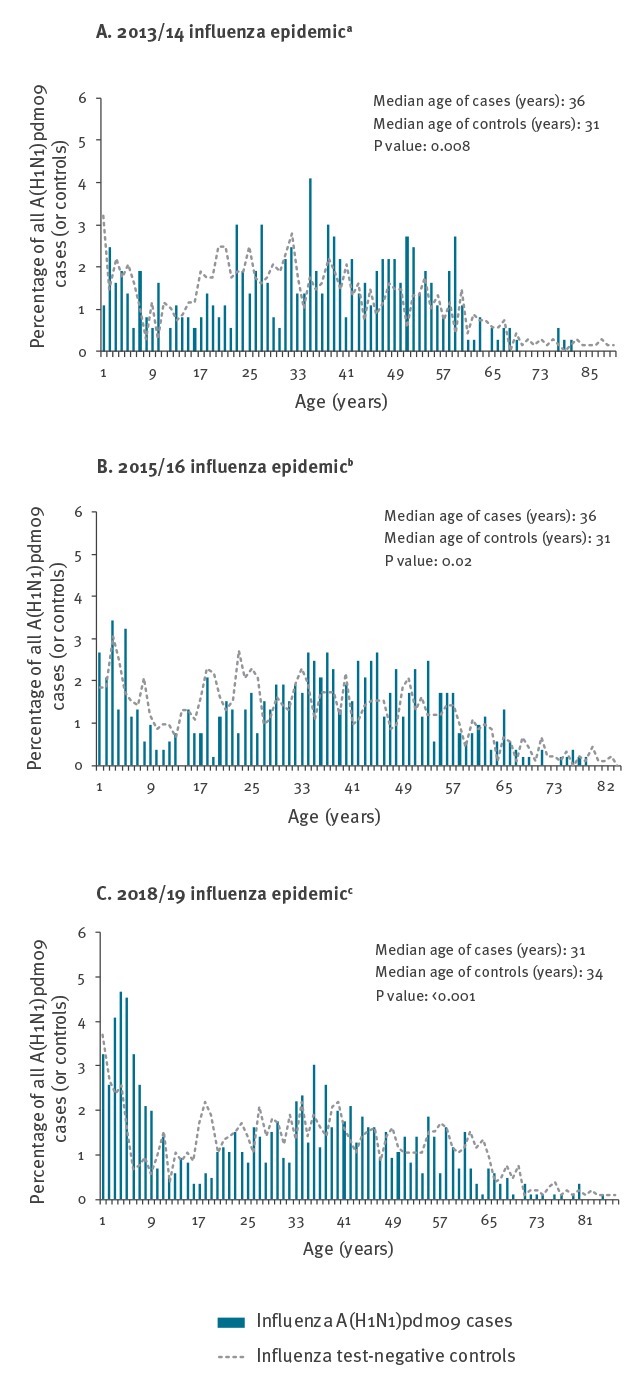
Percentage distribution of unvaccinated influenza A(H1N1)pdm09 cases and controls by single year of age, Canadian Sentinel Practitioner Surveillance Network (SPSN), seasonal influenza A(H1N1)pdm09 epidemics 2013/14, 2015/16, 2018/19

^a^ Span of specimen collection dates for unvaccinated cases (n = 366): 6 November 2013 to 3 April 2014; for unvaccinated controls (n = 683): 1 November 2013 to 29 April 2014.

^b^ Span of specimen collection dates for unvaccinated cases (n = 525): 9 December 2015 to 28 April 2016; for unvaccinated controls (n = 920): 1 November 2015 to 30 April 2016.

^c^ Span of specimen collection dates for unvaccinated cases (n = 858): 1 November 2018 to 14 March 2019; for unvaccinated controls (n = 1050): 1 November 2018 to 18 March 2019.

As shown in Figure 1, the distribution of unvaccinated influenza A(H1N1)pdm09 cases was more highly skewed towards children aged 1–9-years in 2018/19 but with a relative paucity of cases aged 10–19 years during each seasonal A(H1N1)pdm09 epidemic including 2018/19. Shift toward greater involvement of children less than 10 years old was also evident in 2015/16 compared with 2013/14, but became more pronounced in 2018/19. In particular, children between the ages of 3–5 years each comprised the greatest proportion (4% or more) of A(H1N1)pdm09 cases in 2018/19. However, children between the ages of 6–9 years were also more substantially affected, with each single year of age in that grouping newly comprising 2% or more of A(H1N1)pdm09 cases in 2018/19 whereas they had each comprised less than 2% of cases in 2015/16 and 2013/14.

The percentage of all unvaccinated influenza A(H1N1)pdm09 cases belonging to a given single year of age is displayed as a blue bar; the same information for unvaccinated influenza test-negative controls is superimposed as a dotted line to indicate the sampling distribution by age for comparison purposes. The p values displayed are for the comparison between median ages of cases and controls within the same season.

Comparing the age distributions of cases and controls across seasons (not shown) the median age of A(H1N1)pdm09 cases in 2018/19 (31 years) was lower than for cases in 2015/16 and 2013/14 (both 36 years; p < 0.001); whereas, the median age of controls did not significantly differ (or was higher) in 2018/19 (34 years) compared to controls in 2015/16 and 2013/14 (both 31 years; p = 0.04 and 0.11, respectively). Comparing the age distributions of cases versus controls within the same seasons (as shown in Figure 1), the median age of cases in 2018/19 was younger than controls (31 vs 34 years; p < 0.001) whereas cases were older than controls in both 2015/16 and 2013/14 (36 vs 31 years both seasons; p < 0.02 and < 0.01, respectively).

**Figure 2:**
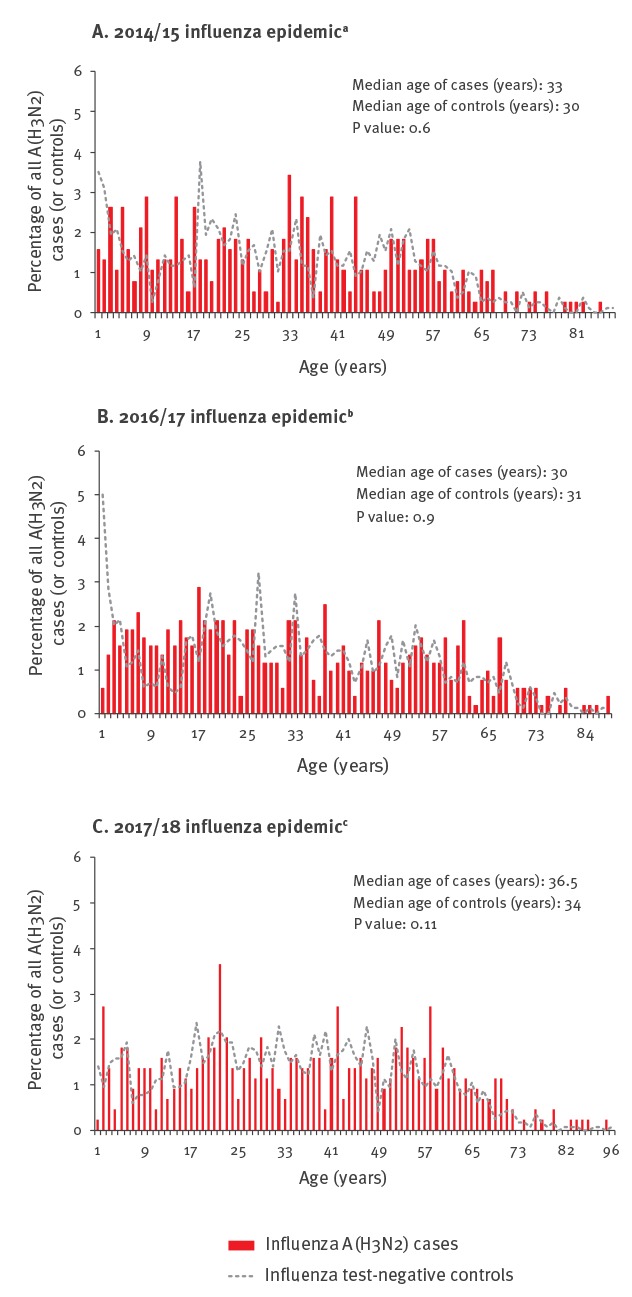
Percentage distribution of unvaccinated influenza A(H3N2) cases and controls by single year of age, Canadian Sentinel Practitioner Surveillance Network (SPSN), seasonal influenza A(H3N2) epidemics 2014/15, 2016/17, 2017/18

^a^ Span of specimen collection dates for unvaccinated cases (n = 380): 5 November 2014 to 1 April 2015; for unvaccinated controls (n = 770): 3 November 2014 to 30 April 2015.

^b^ Span of specimen collection dates for unvaccinated cases (n = 521): 1 November 2016 to 27 April 2017; for unvaccinated controls (n = 840): 1 November 2016 to 29 April 2017.

^c^ Span of specimen collection dates for unvaccinated cases (n = 438): 9 November 2017 to 26 April 2018; for unvaccinated controls (n = 1,139): 1 November 2017 to 30 April 2018.

Influenza A(H3N2) cases were more evenly distributed across the age range (Figure 2). In 2017/18, the median age of influenza A(H3N2) cases was 36.5 years, greater than in 2016/17 (30 years; p = 0.004) and 2014/15 (33 years; p = 0.003). However, a similar pattern was observed in controls, with median age of 34 years in 2017/18 that was greater than in 2016/17 (31 years; p = 0.01) and 2014/15 (30 years; p = 0.001).

The percentage of all unvaccinated influenza A(H3N2) cases belonging to a given single year of age is displayed as a red bar; the same information for unvaccinated influenza test-negative controls is superimposed as a dotted line to indicate the sampling distribution by age for comparison purposes. The p values displayed are for the comparison between median ages of cases and controls within the same season.

The median age of A(H3N2) cases did not significantly differ from controls in any season (all p values > 0.05 as shown in Figure 2).

### Percentage distribution by age category

**Figure 3:**
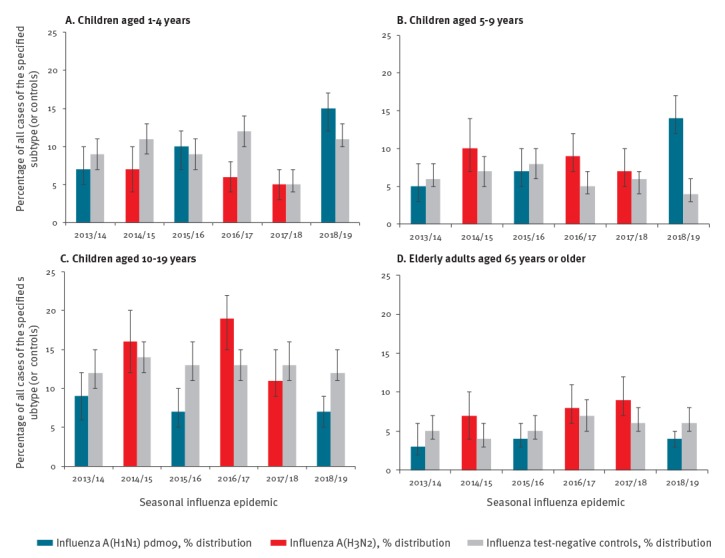
Percentage distribution of unvaccinated cases and controls, paediatric and elderly adult age groups, Canadian Sentinel Practitioner Surveillance Network (SPSN), seasonal influenza epidemics 2013/14–2018/19

In 2018/19, children less than 10 years old comprised a greater proportion of influenza A(H1N1)pdm09 cases than controls (29% vs 16%; p < 0.001), greater also than their share of the general population of SPSN provinces (10%) or their contribution to cases in 2015/16 (17%; p < 0.001) or 2013/14 (12%; p < 0.001). (Figure 3, Table 2 and Supplementary Table S1) [14].

The percentage of all unvaccinated cases of the specified influenza A subtype and all unvaccinated controls within a given season that belonged to the specified age group are displayed. The percentage that were children aged 1–4 years is shown in panel A, children 5–9 years in panel B, children 10–19 years in panel C and adults 65 years and older in panel D. More detailed information, including case distributions among other adult categories, is provided in Table 2.

Blue bars represent A(H1N1)pdm09 case distributions during influenza A(H1N1)pdm09 epidemic seasons (2013/14, 2015/16 and 2018/19), red bars represent A(H3N2) case distributions during influenza A(H3N2) epidemic seasons (2014/15, 2016/17 and 2017/18) and grey bars represent influenza test-negative controls. Associated case/control counts are available in Table 2.

**Table 2:** Percentage distribution of unvaccinated cases and controls by age group, Canadian Sentinel Practitioner Surveillance Network (SPSN), Canada, seasonal influenza epidemics 2013/14–2018/19

	Cases			Controls		
Age group (years)	n	%	95% CI	n	%	95% CI
**Influenza A(H1N1)pdm09 epidemics**						
**2013/14**						
**Total**	**366**			**683**		
1–4	26	**7**	5–10	59	**9**	7–11
5–9	19	**5**	3–8	42	**6**	5–8
10–19	32	**9**	6–12	81	**12**	10–15
20–49	201	**55**	50–60	360	**53**	49–57
50–64	76	**21**	17–25	105	**15**	13–18
≥ 65	12	**3**	2–6	36	**5**	4–7
**2015/16**						
**Total**	**525**			**920**		
1–4	50	**10**	7–12	85	**9**	7–11
5–9	38	**7**	5–10	73	**8**	6–10
10–19	38	**7**	5–10	122	**13**	11–16
20–49	280	**53**	49–58	438	**48**	44–51
50–64	97	**18**	15–22	154	**17**	14–19
≥ 65	22	**4**	3–6	48	**5**	4–7
**2018/19**						
**Total**	**858**			**1050**		
1–4	125	**15**	12-17	119	**11**	10-13
5–9	124	**14**	12-17	47	**4**	3-6
10–19	57	**7**	5-9	131	**12**	11-15
20–49	389	**45**	42-49	487	**46**	43-50
50–64	131	**15**	13-18	200	**19**	17-22
≥ 65	32	**4**	3-5	66	**6**	5-8
**Influenza A(H3N2) epidemics**						
**2014/15**						
**Total**	**380**			**770**		
1–4	25	**7**	4–10	82	**11**	9–13
5–9	38	**10**	7–14	52	**7**	5–9
10–19	59	**16**	12–20	107	**14**	12–17
20–49	166	**44**	39–49	353	**46**	42–49
50–64	67	**18**	14–22	142	**18**	16–21
≥ 65	25	**7**	4–10	34	**4**	3–6
**2016/17**						
**Total**	**521**			**840**		
1–4	29	**6**	4–8	101	**12**	10–14
5–9	49	**9**	7–12	42	**5**	4–7
10–19	97	**19**	15–22	109	**13**	11–15
20–49	211	**40**	36–45	386	**46**	43–49
50–64	91	**17**	14–21	144	**17**	15–20
≥65	44	**8**	6–11	58	**7**	5–9
**2017/18**						
**Total**	**438**			**1139**		
1–4	21	**5**	3–7	62	**5**	4–7
5–9	32	**7**	5–10	65	**6**	4–7
10–19	50	**11**	9–15	152	**13**	11–16
20–49	197	**45**	40–50	578	**51**	48–54
50–64	98	**22**	19–27	209	**18**	16–21
≥ 65	40	**9**	7–12	73	**6**	5–8

CI: confidence interval.

The percentage (%) columns indicate the percentage of total influenza A cases of the specified subtype (or influenza test-negative controls) by seasonal influenza epidemic that belong to the specified age category; these % values are shown in bold for clarity.

The proportion of A(H1N1)pdm09 cases that were children aged 1–4 years increased from 7% in 2013/14 and 10% in 2015/16 to 15% in 2018/19 (p < 0.001 and < 0.01 comparing both prior epidemics to 2018/19, respectively) (Figure 3, Table 2). However, this may reflect increased sampling from that age group, given that their control contribution also increased from 9% in both 2013/14 and 2015/16 to 11% in 2018/19 (p = 0.07 and 0.13, respectively). Overall, the proportion of cases that were children aged 1–4 years in 2018/19 was marginally greater among cases than among controls (15% vs 11%; p = 0.04) with both exceeding the proportion aged 1–4 years in the general population of SPSN provinces (4%) (Supplementary Table S1) [14].

The proportion of A(H1N1)pdm09 cases that were children aged 5–9 years also increased from 5% in 2013/14 and 7% in 2015/16 to 14% in 2018/19 (p < 0.001 comparing both prior epidemics to 2018/19) (Figure 3, Table 2). However, unlike children aged 1–4 years, this pattern was not evident in their control contribution in 2018/19 (4%), with there instead being a slight decline from 2013/14 (6%, p = 0.12) and 2015/16 (8%; p < 0.01). The case contribution by children aged 5–9 years in 2018/19 was thus disproportionate to their control contribution (14% vs 4%; p < 0.001), the latter otherwise approximating their share of the general population (5%) (Supplementary Table S1) [14]. The influenza A(H1N1)pdm09 case contribution by children aged 5–9 years in 2018/19 was also greater than their A(H3N2) case contribution during prior A(H3N2) epidemics (ranging 7–10%; p < 0.05 each comparison) (Figure 3, Table 2).

The case contribution by other age groups in 2018/19 was more comparable to, or below that of, their contribution to controls or to cases and controls during prior seasons (Table 2). In particular, children aged 10–19 years (comprising 11% of the general population) were under-represented among A(H1N1)pdm09 cases relative to controls in 2018/19 (7% vs 12%; p  < 0.001), as well as 2015/16 (7% vs 13%; p < 0.001) and to a lesser extent in 2013/14 (9% vs 12%; p = 0.12) (Figure 3, Table 2,, Supplementary Table S1) [14]. Children 10–19 years old contributed fewer cases across combined A(H1N1)pdm09 epidemics (7%) than combined A(H3N2) epidemics (15%; p < 0.001) or relative to controls combined (13% and 13%, respectively; p < 0.001 both comparisons). Adults aged 65 years and older also contributed less during influenza A(H1N1)pdm09 epidemics combined (4%) compared with A(H3N2) epidemics combined (8%; p < 0.001), while comprising the same overall proportion of controls (6% combined A(H1N1)pdm09 epidemics and 6% combined A(H3N2) epidemics). Each season, however, adults aged 65 years and older were under-represented among outpatient ILI visits relative to their share of the general population (17%) (Supplementary Table S1) [14].

## Discussion

Findings across successive seasons of influenza monitoring by the Canadian SPSN show greater involvement of children aged less than 10 years among outpatient medical visits during the 2018/19 influenza A(H1N1)pdm09 epidemic. In particular, children aged 5–9 years were disproportionately affected, comprising 14% of all cases in 2018/19 which is at least twice that of their contribution during prior A(H1N1)pdm09 epidemics in 2013/14 (5%) and 2015/16 (7%). Over-sampling of children 5–9 years old during the 2018/19 season is unlikely to explain these observations given their contribution to influenza test-negative controls (4%), commensurate with the general population of SPSN provinces (5%) [14].

Moving cohort effects can be followed in relation to major immunological priming events, such as pandemics, to anticipate relative age-related susceptibility during subsequent epidemics. In that regard, our observations in 2018/19 may have an immuno-epidemiological explanation. Infection rates during the 2009 pandemic were very high, especially in children (ca 47% among those aged 5–19 years), resulting in high levels of infection-induced immunity [15,16]. Subsequent seasonal epidemics will have also contributed to population immunity, although influenza infection rates outside of pandemics are generally much lower [17]. Correlating that understanding with our age-related observations, in 2013/14 children aged less than 5 years lacked 2009 pandemic-induced immunity as they were not yet born in 2009; in Canada, this was evident in the lowest sero-protection rates against A(H1N1)pdm09 measured in that age group pre-season in 2013 [16]. Two years later, in 2015/16, this moving cohort of children lacking 2009 pandemic exposure included all children aged 7 years or less; in addition, children under 2 years of age would have missed the opportunity to acquire (or boost) immunity during the 2013/14 influenza epidemic. Finally, in 2018/19, those lacking 2009 pandemic-induced immunity included the expanded group of under 10-year-olds; additionally, children under 5 years lacked exposure to the 2013/14 epidemic and children under 3 years lacked exposure to the 2015/16 epidemic. Overall in 2018/19, compared to prior seasonal influenza epidemics, a greater proportion of children less than 10 years old would have lacked immunity to A(H1N1)pdm09 viruses.

Furthermore, in 2018/19, more than half those born after the 2009 pandemic now attend school (i.e. aged 5–9 years)—a context that did not apply in previous A(H1N1)pdm09 seasonal epidemics and may have enhanced transmission. School children are efficient propagators of infections transmitted by the respiratory or close contact route [18-20]. Facilitating this transmission potential, the mean number of daily contacts increases gradually by age in childhood, peaking between ages 10–19 years [18,19]. Consistent with those epidemiological circumstances, surveillance observations showed that children aged 5–19 years (i.e. school age) contributed disproportionately during influenza A(H3N2) epidemics relative to controls. Younger school age children 5–9 years old also contributed disproportionately during the 2018/19 A(H1N1)pdm09 epidemic, whereas older school age children 10–19 years old were under-represented during all seasonal A(H1N1)pdm09 epidemics. We hypothesise that children aged 5–9 years were disproportionately affected in 2018/19 because for the first time, that well-connected group was fully comprised of children lacking the 2009 pandemic experience. Conversely, older school age children (10–19 years) each season (including 2018/19) were comprised of those who had previously lived through the 2009 pandemic, with greater likelihood of A(H1N1)pdm09 immunity on that basis.

Most contacts of children are of a similar age, but also include siblings and parents and these contacts tend to be of long duration [18,19]. The combination of greater susceptibility and richer contact networks among school-aged children 5–9 years old in 2018/19 may have amplified spread beyond their immediate peers. This may be evident in the higher percentage of influenza test-positive specimens also found among younger children and adults in 2018/19. It may also be evident in the bimodal age distribution of A(H1N1)pdm09 cases with a secondary peak among adults. However, this bimodal age pattern is not evident in the A(H3N2) case distribution, despite the disproportionate involvement of school-aged children during those epidemics also. The appearance of a bimodal peak for A(H1N1)pdm09 cases may instead reflect the relative paucity of cases among those who acquired immunity as children during the A(H1N1)pdm09 pandemic (e.g. those 20 years or younger at the time). It is unknown whether the A(H1N1)pdm09 attack rates in 2018/19 will prove sufficient to arrest or substantially attenuate the moving cohort of paediatric susceptibility we describe following the 2009 influenza pandemic. If not, that susceptibility may next extend to include those older than 10 years who have, on average, the greatest number of effective contacts [18,19], a potentially precarious combination for future A(H1N1)pdm09 epidemics.

Immunological cohort effects may also be expressed in more complex ways across the life span. Priming to the particular influenza viruses of first childhood exposure may leave lasting immunological imprints that can negatively or positively affect responses during subsequent influenza virus encounters [21-23]. Such historic priming effects were hypothesised to explain greater A(H1N1)pdm09 susceptibility among non-elderly adults during recent seasonal epidemics [6,21]. Conversely, immunity generated by early childhood exposure to closely-related ancestral viruses has been hypothesized to explain lower A(H1N1)pdm09 susceptibility decades later among elderly adults during the A(H1N1)pdm09 pandemic [23] and subsequent seasonal A(H1N1)pdm09 epidemics. The timeline for persistence of this pre-existing protection against A(H1N1)pdm09 viruses in elderly adult cohorts requires monitoring. In the meantime, reduced susceptibility to influenza A(H1N1)pdm09 subtype viruses among elderly adults may be negatively correlated with their greater susceptibility to influenza A(H3N2) subtype viruses [24,25], an ecological association that warrants more definitive evaluation.

The SPSN places no age restrictions on influenza testing and includes patients meeting the same ILI testing indication. No relevant system changes were introduced during the study period to account for the age-related shift in influenza A(H1N1)pdm09 cases we observed. Although particular diagnostic assays varied, all test sites are accredited clinical and public health diagnostic and reference laboratories subject to regular proficiency testing programs, conducted at least thrice annually. Either way, assay changes were not applied differentially by age to SPSN specimens. Although children may shed more virus and/or for longer periods [26,27], this is not expected to differ by season and all included specimens were collected within 7 days of ILI onset with the same median interval from ILI onset to specimen collection (3 days) in 2018/19 compared with 2015/16 and 2013/14, overall, stratified by cases/controls and restricted to children 1–9 years of age (data not shown).

To assess age distribution in the absence of vaccine effects, which vary by age, subtype and season [5-10,28], we restricted the analysis to patients who self-reported being unvaccinated. Although we did not account for prior vaccination history, this is highly correlated with current season’s vaccination status [5-10]. In sensitivity analyses, we included patients who self-reported influenza vaccination, and the same age-related patterns were observed (Supplementary Figure S2 and Supplementary Figure S3). This is not unexpected since analyses overall remain driven by the majority of SPSN participants (about two-thirds) [5-10], who did not receive influenza vaccine, similar to the profile in the general population [29]. Inclusive of vaccinated participants, the median ages of both cases and controls were older in all seasons but this is also not unexpected given greater vaccine coverage among elderly adults and those with high-risk conditions targeted by the annual influenza vaccination campaign.

Less than 25% of SPSN participants aged 1–9 years received influenza vaccine each season [5-10], including 2018/19 (data not shown). Between 2014/15 and 2016/17, about half of vaccinated SPSN participants aged 1–9 years for whom the formulation was known were reported to have received live attenuated influenza vaccine (LAIV). However, following changes to LAIV recommendations in the US and Canada [30-32], this proportionate LAIV use fell below 20% among vaccinated participants aged 1–9 years in 2017/18 and 2018/19. Overall, LAIV comprised less than 5% of total influenza vaccine doses distributed by the publicly-funded programme across SPSN provinces in 2018/19. Although there may be some misclassification of vaccine status due to self- or parent/guardian-reporting, it is unlikely that the increased A(H1N1)pdm09 detection we observed among unvaccinated children in 2018/19 could be explained by shedding of LAIV virus among those for whom vaccination occurred but was not recognised, recorded or reported.

Our findings only include patients seeking outpatient care; our system cannot assess patterns among inpatients or those not seeking medical attention. Differences in healthcare seeking or testing behaviours by age and/or epidemic may skew surveillance trends based upon case detection alone. To account for possible age-related sampling variation, we assessed trends among influenza test-negative controls identified according to the same study protocol as for cases, recognising, however, that non-influenza causes of ILI may separately vary by age and season. School-aged children 5–19 years old may have higher influenza attack rates [15,17], but their risk of serious outcomes (such as hospitalisation or death) is lower compared with younger paediatric or other age groups [33-36]. Observations driven by serious outcome surveillance may therefore miss the greater involvement of schoolchildren at the community or outpatient levels. Trends we report in the outpatient setting may not be evident in surveillance systems that include a mix of testing indications or healthcare settings, or for which access to diagnostic testing is targeted by age. Nevertheless, the disproportionate involvement of children less than 10 years of age that we highlight was also reported from Australia during its 2018 A(H1N1)pdm09-dominant epidemic [37] and is discernible in other surveillance systems in Canada for the 2018/19 season, including hospitalisation data [1]. Conversely, the lesser involvement of elderly adults during influenza A(H1N1)pdm09 vs A(H3N2) seasons is evident in fewer long-term care facility outbreaks to date in 2018/19 compared with 2017/18 or 2016/17 in Canada [38].

Limitations of our study include that we used data available season-to-date for 2018/19 (cases spanning to mid-March 2019) but for other years spanning to end-of-season (late April). The timing, peak and mix of influenza and non-influenza causes of ILI may vary each season and their distribution by age may differ toward seasons’ end. This may be especially relevant to the current study given that the involvement of children can be most pronounced during the initial phases of an influenza epidemic [18-20]. However, the seasonal influenza A(H1N1)pdm09 epidemic peak (week 52 2018) has well passed in Canada [1], so that observations summarised about 3 months post-peak (cases spanning to week 11 2019) are likely representative of the A(H1N1)pdm09 epidemic as a whole. Nevertheless, these analyses should be repeated and assessed elsewhere, including regions that may have a different chronology or relative contribution of seasonal influenza A(H1N1)pdm09 circulation since 2009.

## Conclusions

Children less than 10 years old, notably children aged 5–9 years, contributed more to medically-attended cases of influenza A(H1N1)pdm09 illness in Canada in 2018/19 compared to prior seasonal influenza epidemics. Age-related differences likely reflect a combination of immuno-epidemiologic and socio-behavioural factors. Over the next decade, children born after 2009 and lacking pandemic-induced immunity to A(H1N1)pdm09 viruses will be entering the pre-teen and teenage period (10-19 years) associated with the highest social contact rates. The implication for greater transmission during subsequent influenza A(H1N1)pdm09 epidemics warrants ongoing monitoring.

## Supplementary Data

179000 Supplementary Material
