# Supplementary material for: Children under 10 years of age were more affected by the 2018/19 influenza A(H1N1)pdm09 epidemic in Canada: ‎possible cohort effect following the 2009 influenza pandemic
Source: Euro Surveill. 2019 Apr 11;24(15):1900104. doi: 10.2807/1560-7917.ES.2019.24.15.1900104 (PMC6470369; doi:10.2807/1560-7917.ES.2019.24.15.1900104)
Supplement: Supplementary Material [file 19-00104_SKOWRONSKI_Supplement.pdf]

Supplementary Materials

This supplementary material is hosted by *Eurosurveillance* as supporting information alongside the article "*Children under 10 years of age were more affected by the 2018/19 influenza A(H1N1)pdm09 epidemic in Canada: possible cohort effect following the 2009 influenza pandemic*" on behalf of the authors who remain responsible for the accuracy and appropriateness of the content. The same standards for ethics, copyright, attributions and permissions as for the article apply. *Eurosurveillance* is not responsible for the maintenance of any links or email addresses provided therein.

Table of Contents

Supplementary Figure S1. 2018/19 influenza A(H1N1)pdm09 seasonal epidemic curve (unvaccinated cases and controls), Canadian Sentinel Practitioner Surveillance Network (SPSN) 2

Supplementary Figure S2. Percentage distribution of influenza A(H1N1)pdm09 cases and controls overall (vaccinated and unvaccinated) by single year of age, Canadian Sentinel Practitioner Surveillance Network (SPSN), 2013/14, 2015/16, and 2018/19 4

Supplementary Figure S3. Percentage distribution of influenza A(H3N2) cases and controls overall (vaccinated and unvaccinated) by single year of age, Canadian Sentinel Practitioner Surveillance Network (SPSN), 2014/15, 2016/17, and 2017/18 5

Supplementary Figure S1. 2018/19 influenza A(H1N1)pdm09 seasonal epidemic curve (unvaccinated cases and controls), Canadian Sentinel Practitioner Surveillance Network (SPSN)[a]

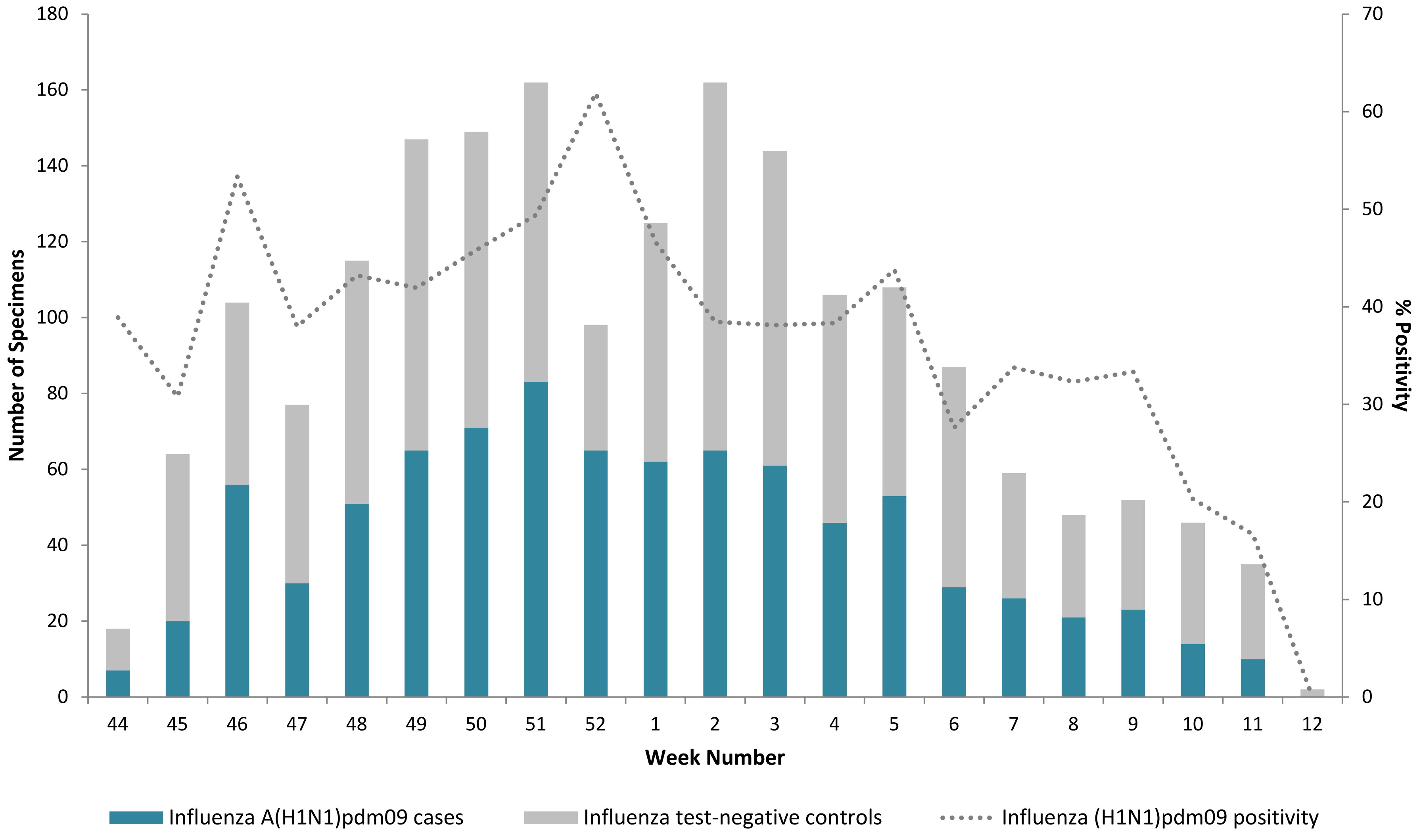

[a] Accrual of influenza A(H1N1)pdm09 cases (n=858) and influenza test-negative controls (n=1050) among unvaccinated participants presenting within 7 days of onset of influenza-like illness. Based on week of specimen collection. Missing specimen collection dates were imputed as the laboratory accession date minus 2 days, the average time between specimen collection and accession dates among specimens with complete information for both values. Data for recent weeks may be incomplete given delays such as in specimen processing and laboratory testing, including subtype characterization.

Supplementary Table S1. Percentage distribution of the general population by age category in provinces contributing to the Canadian Sentinel Practitioner Surveillance Network (SPSN), 2018

SPSN provinces	Age group in years						
	**1 to 4**	**5 to 9**	**10 to 19**	**20 to 49**	**50 to 64**	**65+**	**All ages**
**British Columbia**	184,826	240,976	519,531	2,014,059	1,075,386	912,725	4,947,503
**Alberta**	221,548	276,387	511,892	1,882,019	807,551	551,682	4,251,079
**Ontario**	579,726	761,754	1,638,147	5,789,694	2,983,396	2,424,818	14,177,535
**Quebec**	354,461	460,497	848,691	3,235,655	1,828,069	1,579,277	8,306,650
**Total**	1,340,561	1,739,614	3,518,261	12,921,427	6,694,402	5,468,502	31,682,767
**% of total 2018 population of SPSN provinces, by age group**	4%	5%	11%	41%	21%	17%	100%

Reference: Statistics Canada. Table 17-10-0005-01: Population estimates on July 1st, by age and sex Ottawa: Statistics Canada [Accessed 8 February 2019]. Available from: https://www150.statcan.gc.ca/t1/tbl1/en/tv.action?pid=1710000501

Supplementary Figure S2. Percentage distribution of influenza A(H1N1)pdm09 cases and controls overall (vaccinated[a] and unvaccinated) by single year of age, Canadian Sentinel Practitioner Surveillance Network (SPSN), 2013/14, 2015/16, and 2018/19

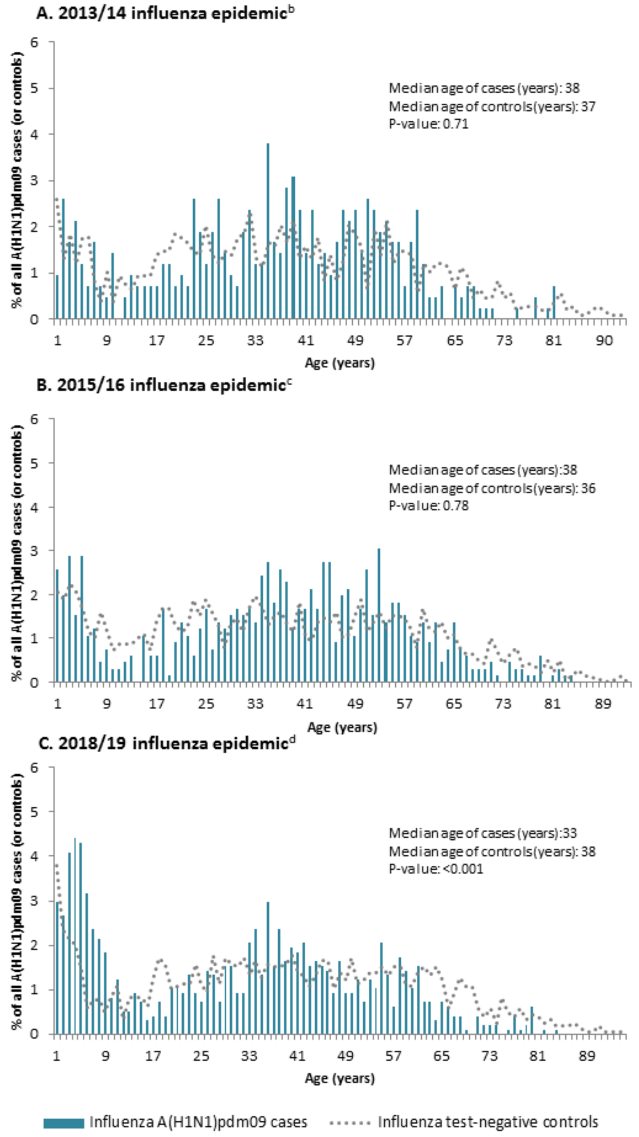

Distributions of cases and controls (including vaccinated[a] and unvaccinated participants) are displayed for influenza A(H1N1)pdm09 epidemics in 2013/14 (A)[b], 2015/16 (B)[c] and 2018/19 (C)[d]. The percentage of all influenza A(H1N1)pdm09 cases belonging to a given single year of age is displayed as a blue bar; the same information for influenza test-negative controls is superimposed as a dotted line to indicate the sampling distribution by age for comparison purposes. The p values displayed are for the comparison between median ages of cases and controls within the same season.

Overall, the median age of A(H1N1)pdm09 cases (overall, vaccinated and unvaccinated) in 2018/19 (33 years) was significantly lower than in 2015/16 and 2013/14 (38 years both seasons; p<0.001 both comparisons); whereas, the median age of controls in 2018/19 (38 years) did not significantly differ from 2015/16 (36 years; p=0.12) or 2013/14 (37 years; p=0.2). The median age of influenza A(H1N1)pdm09 cases within 2015/16 and 2013/14 (38 years both seasons) did not differ from controls (36 years and 37 years, respectively; p=0.78 and p=0.71, respectively) whereas, in 2018/19, A(H1N1)pdm09 cases were significantly younger than controls (33 years vs. 38 years; p<0.001).

---

[a] Self-report (or by parent/guardian) of any influenza vaccine receipt during the season for which the tested specimen was collected.
[b] Span of specimen collection dates for cases (n=421): 6 November 2013 to 3 April 2014; for controls (n=1075): 1 November 2013 to 30 April 2014.
[c] Span of specimen collection dates for cases (n=658): 9 December 2015-28 April 2016; for controls (n=1372): 1 November 2015 to 30 April 2016.
[d] Span of specimen collection dates for cases (n=977): 1 November 2018-14 March 2019; for controls (n=1579): 1 November 2018 to 19 March 2019.

Supplementary Figure S3. Percentage distribution of influenza A(H3N2) cases and controls overall (vaccinated[a] and unvaccinated) by single year of age, Canadian Sentinel Practitioner Surveillance Network (SPSN), 2014/15, 2016/17, and 2017/18

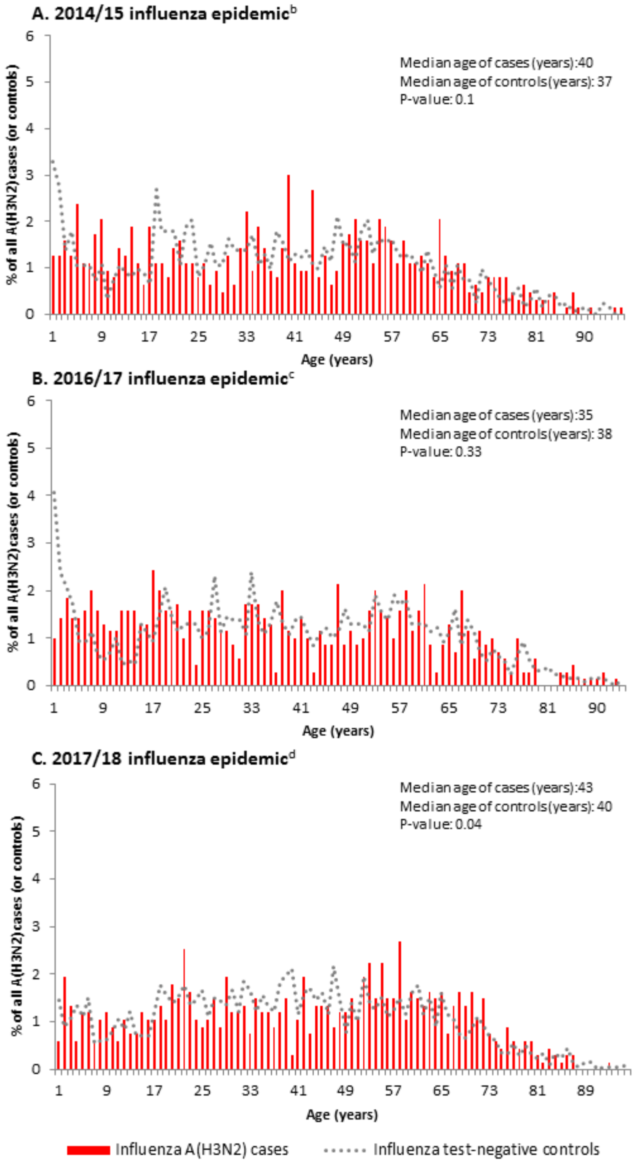

Distributions of cases and controls (including vaccinated[a] and unvaccinated participants) are displayed for influenza A(H3N2) epidemics in 2014/15 (A)[b], 2016/17 (B)[c] and 2017/18 (C)[d]. The percentage of all influenza A(H3N2) cases belonging to a given single year of age is displayed as a red bar; the same information for test-negative controls is superimposed as a dotted line to indicate the sampling distribution by age for comparison purposes. The p values displayed are for the comparison between median ages of cases and controls within the same season.

Although in 2017/18 the median age of influenza A(H3N2) cases (43 years) was greater than in 2016/17 (35 years; p<0.001) and 2014/15 (40 years; p=0.03), this increased age of cases in 2017/18 was paralleled by increased age of controls (40 years) relative to 2016/17 (38 years, p=0.05) and 2014/15 (37 years; p=0.002). Within 2017/18, the older median age of A(H3N2) cases compared to controls was marginally significant (43 years vs. 40 years; p=0.04) but median age of cases and controls was not significantly different within 2016/17 (35 years vs. 38 years; p=0.33) or 2014/15 (40 years vs. 37 years; p=0.1).

---

[a] Self-report (or by parent/guardian) of any influenza vaccine receipt during the season for which the tested specimen was collected.
[b] Span of specimen collection dates for cases (n=634): 5 November 2014 to 22 April 2015; for controls (n=1221): 3 November 2014 to 30 April 2015.
[c] Span of specimen collection dates for cases (n=700): 1 November 2016 to 27 April 2017; for controls (n=1302): 1 November 2016 to 29 April 2017.
[d] Span of specimen collection dates for cases (n=671): 7 November 2017 to 26 April 2018; for controls (n=1792): 1 November 2017 to 30 April 2018.
